# Enhancement of the CRISPR/Cas9-Based Genome Editing System in Lettuce (*Lactuca sativa* L.) Using the Endogenous *U6* Promoter

**DOI:** 10.3390/plants12040878

**Published:** 2023-02-15

**Authors:** Young-Sun Riu, Gwang Hoon Kim, Ki Wha Chung, Sam-Geun Kong

**Affiliations:** 1 Department of Biological Sciences, Kongju National University, Gongju-si 32588, Republic of Korea; 2 Biotechnology Research Institute, Kongju National University, Gongju-si 32588, Republic of Korea

**Keywords:** chloroplast movement, CRISPR/Cas9, *Lactuca sativa* L., phototropin2, transgene-free editing, *U6* promoter

## Abstract

The CRISPR/Cas9 system has been widely applied as a precise gene-editing tool for studying gene functions as well as improving agricultural traits in various crop plants. Here, we optimized a gene-editing system in lettuce (*Lactuca sativa* L.) using the endogenous *U6* promoter and proved that the *PHOT2* gene is a versatile target gene. We isolated the *LsU6-10* promoter from 10 *U6* snRNA genes identified from the lettuce genome database for comparison with the *AtU6-26* promoter that has been used to drive sgRNAs in lettuce. Two CRISPR/Cas9 vectors were constructed using the *LsU6-10* and *AtU6-26* promoters to drive sgRNA361 to target the *PHOT2* gene. The chloroplast avoidance response was defective in lettuces with biallelic mutations in the targeted *PHOT2* gene, as in the *Arabidopsis phot2* mutant. The *PHOT2* gene mutations were stably heritable from the R0 to R2 generations, and the high gene-editing efficiency enabled the selection of transgene-free lines in the R1 generation and the establishment of independent *phot2* mutants in the R2 generation. Our results suggest that the *LsU6-10* promoter is more effective than the *AtU6-26* promoter in driving sgRNA for the CRISPR/Cas9 system in lettuce and that *PHOT2* is a useful target gene to verify gene editing efficiency without any detrimental effects on plant growth, which is often a consideration in conventional target genes.

## 1. Introduction

Genome-editing technologies enable precise changes to genomes by targeting specific DNA. Given the advantages of genome editing technology, genome-editing in plants is widely utilized not only to characterize gene functions but also to improve agricultural traits [1,2,3]. To date, a number of genome-editing technologies have been successfully developed including zinc-finger nucleases (ZFNs), transcription activator-like effector nucleases (TALENs), and the clustered regularly interspaced short palindromic repeats (CRISPR)/CRISPR-associated protein 9 (Cas9) system [4,5,6,7,8]. These genome-editing techniques are based on the processes of double-strand breaks (DSBs) in specific DNA followed by DNA repair systems via nonhomologous end-joining (NHEJ) or homologous recombination (HR) in vivo [1,4,9]. Predominantly, DSBs are repaired by the NHEJ pathway, which is prone to errors, by which insertion or deletion (indels) of one or more nucleotides occurs at the cleavage site to cause gene knockout [1,9].

The CRISPR/Cas9 system has been successfully applied to generate new plants with desirable features by gene editing in various plant species, including model plants and crop plants, because of its simplicity, high efficiency and inexpensive preparation compared to ZFNs and TALENs [10,11,12,13,14]. The CRISPR/Cas9 system consists of two main components: Cas9 endonuclease, which is required for DNA double-strand breaks at the desired loci, and small guide RNA (sgRNA), which binds Cas9 endonuclease to a specific target DNA site [1,15]. In the plant CRISPR/Cas9 system, the Cas9 endonuclease is generally expressed by strong ubiquitous promoters, such as the *Cauliflower mosaic virus (CaMV) 35S* promoter, and the *ubiquitin* promoter, and sgRNAs are usually expressed by RNA polymerase-III (Pol III) promoters, such as *U6* and *U3* [13,16].

The *U6* promoter has been commonly used to drive high expression of sgRNAs in plants. The *U6* promoter has a highly conserved transcription initiation site starting with a guanine nucleotide, which helps to improve the homogeneity of the transcribed sgRNA molecule and to reduce off-target effects [2,17]. For these reasons, the *Arabidopsis U6 (AtU6)* promoter and rice (*Oryza sativa*) *U6* (*OsU6*) promoter have been widely used to express sgRNAs heterologously in many plant species [11,18,19,20]. In general, in the CRISPR/Cas9 system, the *AtU6* promoter is used in dicot plants, and the *OsU6* promoter is applied in monocot plants [1,13,16]. However, heterologous applications of the *U6* promoters are limited only to closely related plant species [21]. Recently, in several plant species, such as wheat, soybean, chicory, cotton and grape, it has been shown that endogenous *U6* promoters drive high sgRNA expression and improve editing efficiency [22,23,24,25,26,27]. Therefore, the use of endogenous *U6* promoters should be considered to optimize the efficiency of CRISPR/Cas9-mediated genome editing in crop plants.

Phytoene desaturase (PDS) is an essential plant carotenoid biosynthesis enzyme, and a null mutant of this gene exhibits an albino phenotype [28]. With this visible phenotypic characteristics, the *PDS* gene is widely adopted as a target gene when evaluating the genome editing efficiency of the CRISPR/Cas9 system in various plant species [22,24,26,29]. Phototropins (phot1 and phot2 in angiosperms) are blue light receptors that mediate a range of blue light responses, including chloroplast movement, phototropism, stomatal opening, and leaf flattening [30]. In *Arabidopsis thaliana*, both phot1 and phot2 mediate the chloroplast accumulation response under low-intensity blue light conditions. On the other hand, phot2 alone mediates the chloroplast avoidance response under high-intensity blue light conditions [31,32,33]. Importantly, the *phot2* mutant has a very distinct phenotype that shows a constitutive chloroplast accumulation response even under high-intensity blue light conditions [31,33]. Therefore, the *PHOT2* gene, similar to the *PDS* gene, could be a useful target gene to verify the editing efficiency of the CRISPR/Cas9 system in plants.

Lettuce (*Lactuca sativa* L.) is one of the most grown vegetable crops worldwide and has high economic value [34]. Lettuce is rich in vitamins, carotenoids, folic acid, and antioxidants that are beneficial to human health [34,35]. In particular, red leaf lettuce accumulates a high concentration of anthocyanin pigments, which have excellent antioxidant effects [34,36]. Recently, CRISPR/Cas9-based gene editing has been successfully demonstrated in lettuce, which is a model vegetable crop with a variety of characteristics. However, the *AtU6-26* promoter has been heterologously applied to drive sgRNAs for the CRISPR/Cas9 system in lettuce [37,38,39]. Therefore, in this study, we aimed to establish an efficient CRISPR/Cas9 genome editing system in lettuce using an endogenous *LsU6-10* promoter for high sgRNA expression and the *PHOT2* gene as a target gene facilitating phenotype-based selection. Using an efficient genome editing system, we also aimed to generate transgene-free lettuce *phot2* mutants through genetic segregation.

## 2. Results

### 2.1. Identification of U6 Promoters in Leaf Lettuce

The *AtU6* promoter has been commonly used to drive sgRNAs in dicots [16]. In a previous study, although the use of the *AtU6-26* promoter to induce sgRNA expression resulted in successful gene editing in lettuce [37,38], the efficiency of genome editing was not evaluated in the CRISPR/Cas9 system using the heterologous *AtU6-26* promoter. Therefore, in comparison with the heterologous *AtU6-26* promoter, we attempted to use the endogenous *U6* promoter to optimize the CRISPR/Cas9 system in lettuce. 

**Figure 1:**
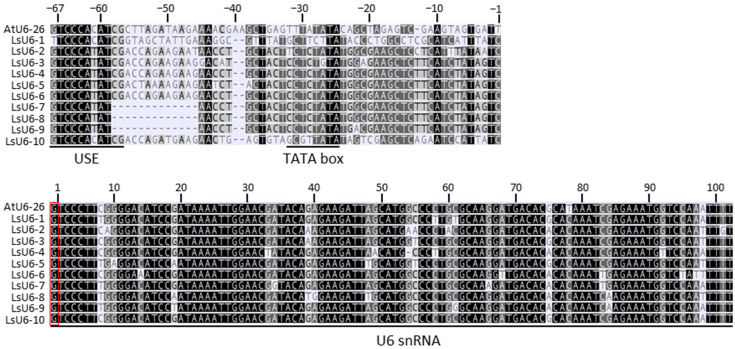
Multiple sequence alignment of lettuce *U6* and *Arabidopsis U6-26 snRNA* genes. Highly conserved portions of the sequence are shown in black. Upstream sequence element (USE), TATA-box and *U6* small nuclear RNA (snRNA) sequences are underlined. The transcription start sites (+1) are indicated by a red box. The candidate lettuce *U6* genes were named LsU6-1 for LOC111919320, LsU6-2 for LOC111918078, LsU6-3 for LOC111911168, LsU6-4 for LOC111911169, LsU6-5 for LOC111918392, LsU6-6 for LOC111912034, LsU6-7 for LOC111919806, LsU6-8 for LOC111914804, LsU6-9 for LOC111919805, and LsU6-10 for LOC111913621. Note that LsU6-10 showed the highest similarity to AtU6-26.

Ten lettuce *U6 snRNA* genes were obtained from the lettuce genome database by BLAST using the *AtU6-26 snRNA* gene sequence. The 10 lettuce *U6 snRNA* gene sequences showed very high similarity to the *AtU6-26 snRNA* gene sequence in their overall transcript sequences starting with a guanine nucleotide as the transcription initiation site. In addition, the promoter regions contained the upstream sequence elements (USEs) required for transcription and the conserved elements of the TATA-like boxes. However, the promoter regions without the two elements were very different from not only the *AtU6-26* promoter region but also each other (Figure 1). Among the 10 lettuce *U6 snRNA* genes, the promoter of the *LOC111913621* gene (named *LsU6-10*) was the most similar to the *AtU6-26* gene. Therefore, the *LsU6-10* promoter was isolated as an endogenous promoter to induce sgRNA expression, and its potential use was examined for CRISPR/Cas9 gene editing in lettuce (see below).

### 2.2. Plasmid Construction for the CRISPR/Cas9-Mediated Gene Editing System in Lettuce

**Figure 2:**
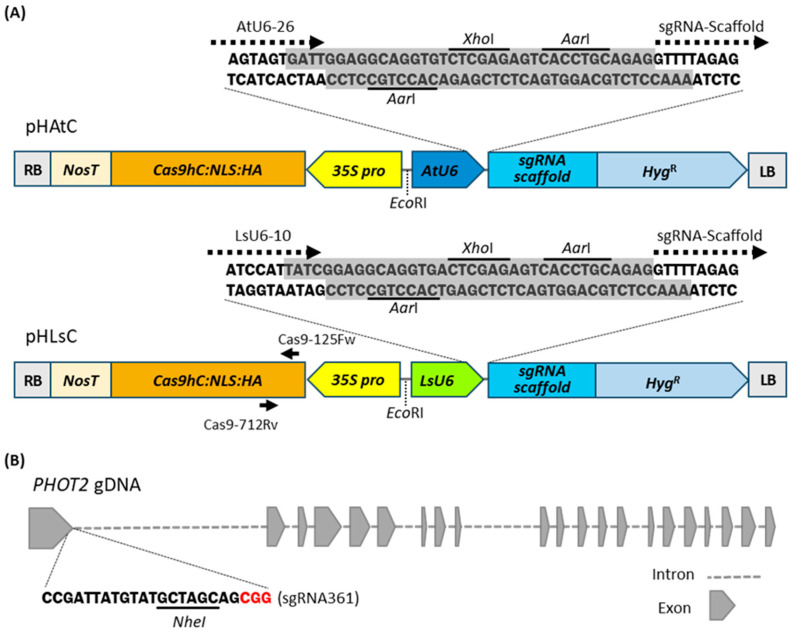
Schematic diagrams of CRISPR/Cas9 expression vectors and sgRNA targeting site in *PHOT2* gene. (**A**) Schematic diagrams of CRISPR/Cas9 expression vectors used for *Agrobacterium*-mediated lettuce transformation. In pHAtC, a single guide RNA (sgRNA) cassette is driven by the *Arabidopsis U6-26* promoter (AtU6), and in pHLsC, the sgRNA cassette is driven by the *Lactuca sativa U6-10* promoter (LsU6). Cas9-125Fw and Cas9-712Rv indicate a primer set used to confirm transgene integrations. NosT, nopaline synthase (NOS) terminator; Cas9hc:NLS:HA, human codon-optimized Cas9 expressing cassette; 35S pro, *CaMV 35S* promoter; Hyg^R^, Hygromycin resistance gene cassette; LB, left border; RB, right border. The *Aar*I recognition site (CACCTGC) and *Xho*I recognition site (CTCGAG) are underlined. (**B**) A schematic diagram of the sgRNA361 targeting site in the genomic region of *PHOT2.* The PAM motif (CGG) is shown in red; the *Nhe*I recognition site (GCTAGC) is underlined.

For CRISPR/Cas9 gene editing in lettuce, we modified the pHAtC vector that was originally developed for *Arabidopsis* gene editing using the *AtU6-26* promoter to drive sgRNAs and the *CaMV 35S* promoter to drive Cas9 expression [40]. The DNA fragment of the *AtU6-26* promoter in pHAtC was replaced with the corresponding DNA fragment of the *LsU6-10* promoter and named pHLsC (Figure 2A). Each vector allows easy insertion of a 20-bp sgRNA seed sequence with two *Aar*I between the *U6* promoter and the sgRNA scaffold. 

To determine the gene editing efficiency of CRISPR/Cas9 expression vectors in lettuce, the blue light receptor *PHOT2* gene was set as the target gene. The sgRNA was designed to target the first exon of *PHOT2* (NCBI Reference Sequence: XM_023876578.2) using the CRISPOR program [http://crispor.tefor.net/crispor.py (accessed on 17 May 2021)]. The designed sgRNA361 contained the *Nhe*I restriction site, which was used for the PCR-based restriction enzyme (PCR/RE) digestion assay to screen for indel mutations (Figure 2B). The double-stranded DNA fragment of the sgRNA361 cassette was inserted into the pHAtC vector and the pHLsC vector using the *Aar*1 restriction sites to produce pHAtC-PHOT2 and pHLsC-PHOT2, respectively. The completed vectors were transformed into the cotyledon cells of red leaf lettuce through *Agrobacterium*-mediated transformation. Beginning two months later, hygromycin-resistant transgenic plants (R0) were regenerated from independent hygromycin-resistant calli. The transgenic plants were named as phot2-At for transgenic plants transformed with pHAtC-PHOT2, and phot2-Ls for transgenic plants transformed with pHLsC-PHOT2, respectively. For further molecular and physiological analyses, 21 phot2-At and 22 phot2-Ls transgenic lettuce plants were independently isolated in the R0 generation (Table 1).

### 2.3. The LsU6 Promoter Has Higher Efficiency Than the AtU6 Promoter in CRISPR/Cas9-Mediated Gene Editing in Lettuce

**Figure 3:**
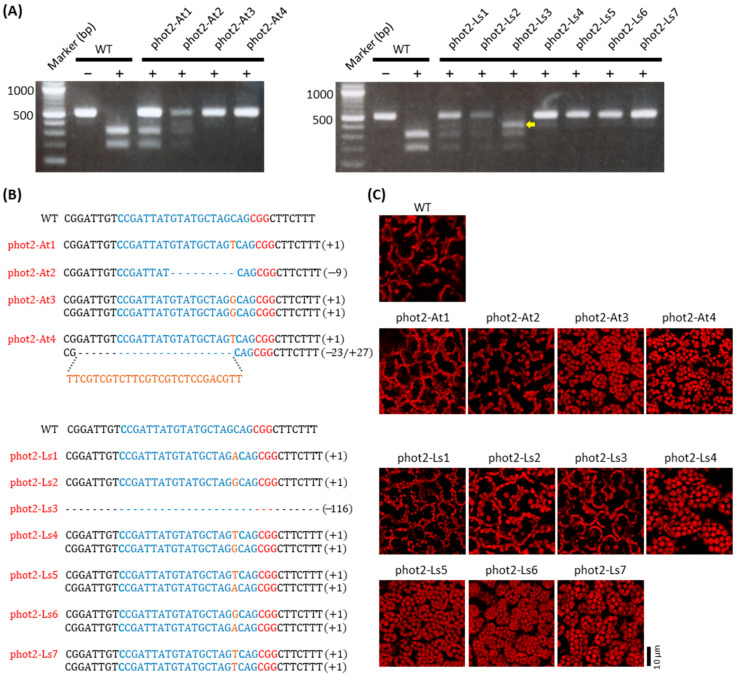
CRISPR/Cas9-induced targeted mutagenesis of *LsPHOT2* in lettuce. (**A**) Detection of mutations at the sgRNA361 targeting site of R0 Atphot2 and Lsphot2 transgenic lines. Note that the PCR products of the *phot2* mutant lines were resistant to *Nhe*I digestion. ‘+’ means *Nhe*I treatment; ‘−’ means without *Nhe*I treatment. The yellow arrow indicates an undigested DNA fragment originating from a 166-bp deletion (see details in B). The marker on the left of each photo indicates the 100 bp DNA ladder. (**B**) Sequence-based detection of *phot2* mutations in R0 phot2-At and phot2-Ls transgenic lines. The PAM motif is shown in red, sgRNA361 target sequences are shown in blue, insertions are highlighted in orange, and dashes indicate deletions. (**C**) Chloroplast positioning under a high-intensity blue light condition in R0 lettuce *phot2* mutant lines generated by CRISPR/Cas9-induced targeted mutagenesis. The leaves of WT and R0 phot2-At and phot2-Ls lines were set on an agar plate containing 0.5% gellan gum and irradiated with high-intensity blue light (50 μmol m^−2^ s^−1^) for 2 h. Chloroplast positioning was observed using a confocal laser scanning microscope. Scale bar = 10 µm.

We used a PCR/RE digestion assay to determine whether indel mutations were present at the sgRNA361 target site on the *PHOT2* gene in phot2-At and phot2-Ls transgenic lettuce plants. The 520-bp genomic fragments were amplified by PCR using the specific primer set (phot2-44Fw and phot2-intron_Rv) against genomic DNA templates extracted from the leaf tissue of each transgenic lettuce plant. Thereafter, the PCR products were treated with *Nhe*I, and the cutting patterns were confirmed through electrophoresis (Figure 3A). In the WT, 520-bp DNA fragments were amplified and digested into ca. 314 bp and 206 bp DNA fragments by *Nhe*I treatment, whereas the PCR fragments were amplified in ca. 520-bp DNA sizes from the genomic DNA of phot2-At and phot2-Ls transgenic lettuce plants, and *Nhe*I digestion was partial or did not occur (Figure 3A). The monoallelic mutations showed a total of three bands with two digested bands (ca. 314 bp and 206 bp DNA fragments) and one undigested band (520 bp DNA fragment) in phot2-At1, 2 and phot2-Ls1, 2, 3 (Figure 3A). The biallelic mutations showed only one undigested band in phot2-At3, 4, and phot2-Ls4, 5, 6, 7 (Figure 3A). PCR/RE digestion assays suggested that gene mutation was efficiently induced by our CRISPR/Cas9-medited gene editing system using both pHAtC-PHOT2 and pHLsC-PHOT2 vectors. 

Next, we performed cloning and sequencing to confirm the indel patterns in phot2-At and phot2-Ls lettuce plants showing either partial or undigested bands following *Nhe*I treatment. The sequencing results showed that mutations with short nucleotide insertions or deletions were generated at the sgRNA361 target sites on the *PHOT2* gene in phot2-At and phot2-Ls transgenic lettuce plants (Figure 3B; Table 1). Mutation efficiency was much higher in phot2-Ls transgenic lettuce plants (16 plants out of 22 plants, 73%) than in phot2-At transgenic lettuce plants (10 plants out of 21 plants, 48%) (Table 2). Interestingly, the biallelic mutation type was much more frequently found in phot2-Ls transgenic lettuce plants than in phot2-At transgenic lettuce plants: the mutation type was 20% monoallelic mutation and 19% biallelic mutation for phot2-At transgenic lettuce plants and 32% monoallelic mutation and 41% biallelic mutation for phot2-Ls transgenic lettuce plants (Table 2). Consequently, our results suggest that the use of the lettuce endogenous *LsU6-10* promoter increases gene-editing efficiency in lettuce compared to that of the heterologous *AtU6-26* promoter.

### 2.4. Lettuce Phot2 Mutants Are Defective in the Chloroplast Avoidance Response

The indel mutations in phot2-At and phot2-Ls transgenic lettuce plants induced frameshifts causing premature termination codons in phot2 translation (Figure 3B). The *Arabidopsis phot2* mutant is defective in the chloroplast avoidance response by which chloroplasts accumulate at the cell surface even under high-intensity blue light conditions [31,32,33]. Therefore, we observed the chloroplast avoidance response in the palisade mesophyll cells of phot2-At and phot2-Ls transgenic lettuce plants after the leaves were irradiated with high-intensity blue light (50 μmol m^−2^ s^−1^) for 2 h (Figure 3C). In the WT, chloroplasts were positioned along the anticlinal sides of palisade mesophyll cells, indicating that the avoidance response was actively induced under the high-intensity blue light condition. Similarly, the chloroplast avoidance response was effectively observed in the palisade mesophyll cells of both phot2-At lines (e.g., phot2-At1, phot2-At2, etc.) and phot2-Ls lines (e.g., phot2-Ls1, phot2-Ls2, phot2-Ls3, etc.) with monoallelic mutations (Figure 3B,C; Table 1). In contrast, chloroplasts accumulate at the palisade mesophyll cell surface of phot2-At (e.g., phot2-At3, phot2-At4, etc.) and phot2-Ls (e.g., phot2-Ls4, phot2-Ls5, phot2-Ls6, phot2-Ls7, etc.) transgenic lettuce plants with biallelic mutations, indicating that these phot2-At and phot2-Ls plants are defective in avoidance response (Figure 3B,C; Table 1). Consistent with the genotype results and the previously described *phot2* mutant in *Arabidopsis* (Figure 3B; [31,32,33]), phot2-At and phot2-Ls transgenic lettuce plants harboring biallelic indel mutations in the *PHOT2* gene were completely defective in the chloroplast avoidance response, similar to the *phot2* mutant.

### 2.5. Selection of Transgene-Free and Genome-Edited Phot2 Mutant

Next, we attempted to select transgene-free and stable *phot2* homozygous mutant lines from phot2-Ls transgenic lettuce plants by eliminating the T-DNA transgene. For the experiment, we chose two independent phot2 mutant lines in the R1 generation, the phot2-Ls2 line with a monoallelic mutation (guanine nucleotide insertion) and the phot2-Ls5 line with a biallelic mutation (thymidine and adenine nucleotide insertion) (Figure 3, Table 1). 

**Figure 4:**
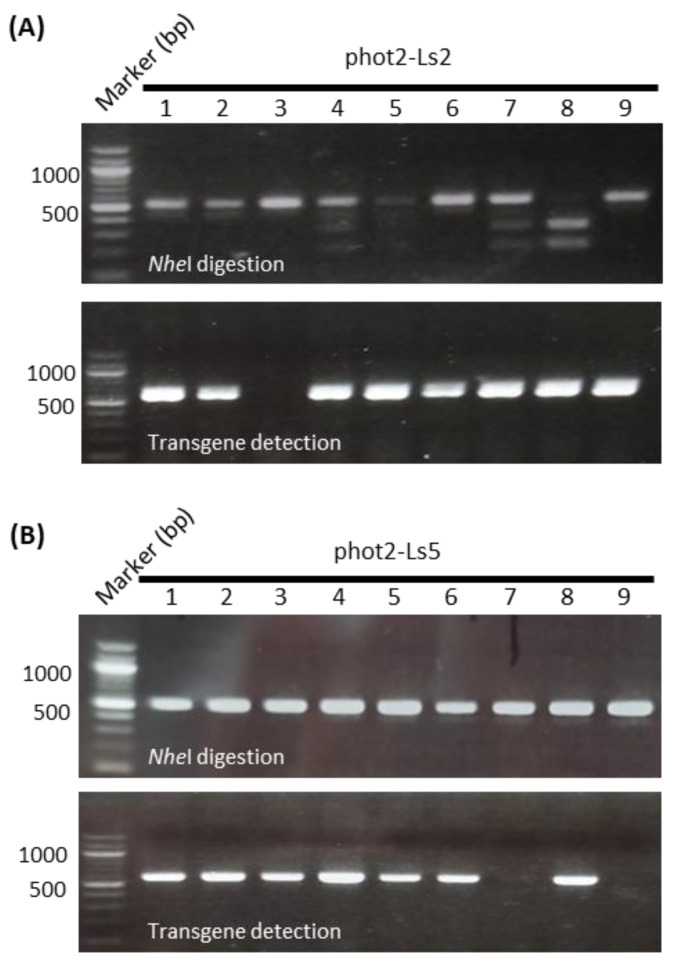

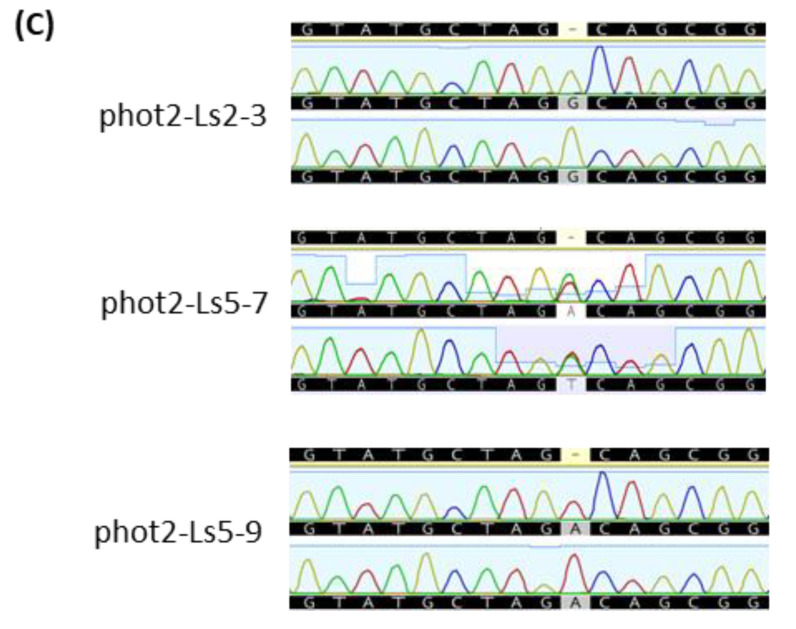
Screening of germline transmission of *phot2* mutations without transgene in R1 phot2-Ls lines. (**A**,**B**) Isolations of stable *phot2* mutant lines without transgene from the phot2-Ls in the R1 generation. phot2-Ls2-3 (**A**) and phot2-Ls5 (**B**) were selected as the representative lines of monoallelic and biallelic mutants, respectively. *Nhe*1 digestion (upper panel): *Nhe*1 digestions of PCR products to detect indel mutations at the sgRNA361 targeting site. Other details are the same as those shown in Figure 3A. Transgene detection (lower panel): PCR products for transgene using a Cas9-specific primer set (see Figure 2A). Note that the lines phot2-Ls2-3, phot2-Ls5-7, and phot2-Ls5-9 are resistant to *Nhe*I digestion and have no transgene. (**C**) Sequence-based detection of phot2 mutations in R1 phot2-Ls2 and phot2-Ls5 lines. Other details are the same as those shown in Figure 3B.

First, the R1 siblings of phot2-L2 and phot2-L5 lines were examined for the insertional mutations by PCR/RE digestion assay using *Nhe*I digestion and sequence-based detection and transgene detection by PCR using the specific primer set for Cas9 (Cas9-125Fw and Cas9-712Rv) (Figure 4). As expected, both phot2-Ls2 and phot2-Ls5 lines were genetically segregated on not only the insertional mutation pattern but also transgene integration. As a result, a line of phot2-Ls2-3 was successfully selected from nine independent R1 phot2-L2 lines, satisfying both conditions of no *Nhe*1 digestion and no transgene detection (Figure 4A). Similarly, two lines of phot2-L5-7 and phot2-L5-9 were successfully selected from nine independent R1 phot2-Ls5 lines, in which *Nhe*1 digestion did not occur in all lines, but transgene-free was only confirmed in two lines, phot2-Ls5-7 and phot2-Ls5-9 (Figure 4B). DNA sequencing further indicated that phot2-Ls2-3 and phot2-Ls5-9 were homozygous mutants with one nucleotide insertion of guanine or adenine, respectively. In contrast, phot2-Ls5-7 was a heterozygous mutant with a biallelic mutation of adenine and thymidine, as in the R1 generation (Figure 3B and Figure 4C). From the results, it was confirmed that both phot2-Ls2-3 and phot2-Ls5-9 lines were transgene-free, in which gene-edited mutations of one nucleotide insertion were safely transmitted from the R0 to R1 generations (Figure 4A,B). 

**Figure 5:**
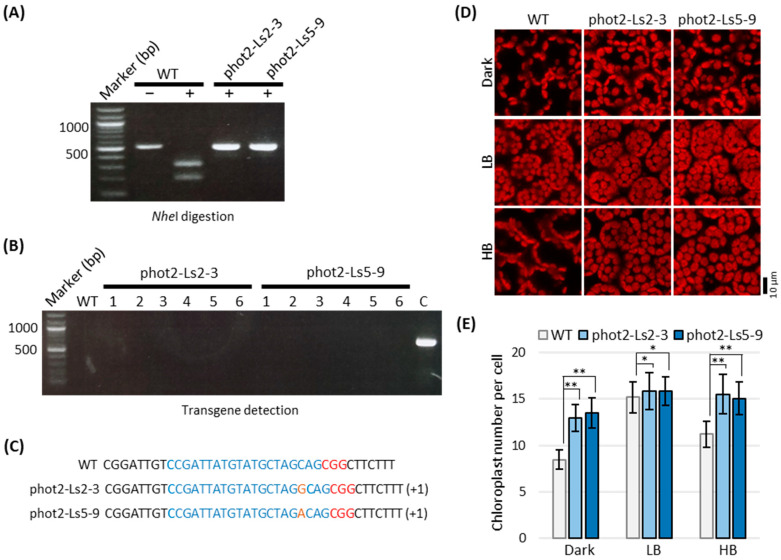
Germline transmission of *phot2* mutations in phot2-Ls lines to the R2 generation. (**A**) Detection of *phot2* mutations at the sgRNA361 targeting site of the phot2-Ls2-3 and phot2-Ls5-9 lines. Other details are the same as those shown in Figure 3A. Note that the lines are resistant to *Nhe*I digestion. (**B**) Confirmation of transgene-free in R2 Lsphot2-3 and phot2-Ls5-9 lines. PCR detection was carried out using a Cas9-specific primer set (see Figure 2A). (**C**), PCR control using phot2-Ls2-1 (Figure 4A). (**C**) Sequencing-based detection of *phot2* mutations in the R2 phot2-Ls2-3 and phot2-Ls5-9 lines. Other details are the same as those shown in Figure 3B. Note that the genome-edited lines phot2-Ls2-3 and phot2-Ls5-9 are transgene-free and resistant to *Nhe*I digestion. (**D**,**E**) Chloroplast movement in the R2 phot2-Ls2-3 and phot2-Ls5-9 lines. Chloroplast positioning (**D**) and chloroplast number per cell (**E**) were investigated in the palisade mesophyll cells of leaves of 4-week-old phot2-Ls2-3 and phot2-Ls5-9 lines under different intensities of blue light. Dark, dark adaptation for 12 h; LB, low-intensity blue light (2 μmol m^−2^ s^−1^) for 2 h; HB, high-intensity blue light (50 μmol m^−2^ s^−1^) for 2 h. Note that the phot2-Ls lines are defective in chloroplast avoidance response as well as dark-positioning. Scale bar in D = 10 µm. Data in E represent the mean ± SE (*n* = 15 cells). Asterisks indicate statistical significance detected by Student’s *t*-test: * *p* > 0.05, not statistically significant; ** *p* < 0.0001, statistically significant.

Phot2-Ls2-3 and phot2-Ls5-9 lines in the R2 generation were further confirmed for the safe transmittance of mutations and the *phot2* mutant phenotype. Analyses of PCR/RE digestion and PCR-based transgene detection and DNA sequencing consistently suggested that the phot2-Ls2-3 and phot2-Ls5-9 lines were transgene-free and homozygous, harboring one nucleotide insertional mutation (guanine or adenine nucleotides) in the first exon of the *PHOT2* gene, which was safely transmitted from the R0 to R2 generations (Figure 5A–C). Consistent with genotypic analysis, the phot2-Ls2-3 and phot2-Ls5-9 lines were specifically defective in the chloroplast avoidance response as well as in dark positioning but were normal in the chloroplast accumulation response, as previously described in the Arabidopsis *phot2* mutant (Figure 5D,E; [31,32,33,41]).

## 3. Discussion

Gene editing is an essential technology for studying gene function and improving productivity and functionality not only in model plants but also in various crop plants [42]. In particular, the CRISPR/Cas9 system has been widely used due to its advantages of simple design, low cost, and high efficiency. The success of genome editing using the CRISPR/Cas9 system is highly dependent on the expression levels and tissue-specific expression of sgRNA and Cas9. In plants, sgRNAs have frequently been driven in various plants by using *Arabidopsis* and rice *U6* promoters. However, heterologous *U6* promoters are less effective in some species. This problem has been overcome by driving sgRNA expression using endogenous *U6* promoters in wheat, soybean, chicory, cotton, and grape [22,23,24,25,26,27]. The *AtU6-26* promoter and *35S* promoter have been commonly used to drive sgRNA and Cas9 expression for the CRISPR/Cas9 system in lettuce [37,38,39]. Therefore, in this study, we verified the application of the endogenous *U6* promoter to improve the CRISPR/Cas9 system in lettuce.

On the basis of DNA sequence similarity with the *Arabidopsis U6* promoter, 10 lettuce *U6* promoters, designated LsU6-1 to -10, were isolated from the lettuce genome (Figure 1). Among these, the *LsU6-10* promoter, which was the most similar to the snRNA region of *AtU6-26*, was chosen to drive sgRNA, and a CRISPR/Cas9-mediated genome editing vector system targeting the *PHOT2* gene was constructed (Figure 2). The results obtained from the R0 generation to the R2 generation showed that the CRISPR/Cas9-mediated gene editing system using the endogenous lettuce *U6-10* promoter significantly improved the mutation efficiency targeting the *PHOT2* gene compared with the heterologous *AtU6-26* promoter (Table 1). DNA sequence alignment between *LsU6* promoters and the *AtU6-26* promoter indicated that the snRNA genes had a very high identity of over 91% in the transcribed region but a relatively low identity of approximately 54% in the promoter region, including the conserved USE and TATA box elements (Figure 1). Therefore, the different promoter activity between the *U6-10* and *At6-26* promoters in lettuce could result from the different binding activities of transcription factors to the regulatory elements. 

The efficiency of gene editing was much higher from sgRNA361 expression by the *LsU6-10* promoter compared with the *AtU6* promoter (mutation rate: 78% for *LsU6-10* promoter vs 48% for *At6-26* promoter); furthermore, the mutation patterns on the *PHOT2* gene were more diverse in phot2-Ls lines than phot2-At lines in the R0 generation (Figure 3B; Table 2). In particular, biallelic mutations were much more frequently found in the phot2-Ls lines, with a total of four indel patterns (41% for biallelic vs 32% monoallelic), than in the phot2-At lines, with a total of two indel patterns (19% for biallelic vs 29% monoallelic). A high ratio of biallelic mutants was able to be utilized directly for analyses of gene function and phenotype, which is very beneficial to save time and labor in plant genome editing. The efficiency of CRISPR/Cas9-induced mutations is also advantageously utilized to establish transgene-free lines through genetic segregation (Figure 4A,B and Figure 5B). As a result, the use of the endogenous *U6* promoter once again proves to be a very useful tool for increasing efficiency in gene editing in lettuce. In addition, it was demonstrated that the gene-edited mutants using the new vector system were stably inherited to the next generation (Figure 3, Figure 4 and Figure 5; Table 1).

The choice of target gene is important to obtain convenience, especially in experiments to verify the efficiency of genome editing. Thus, when the gene is knocked out, the easily observable albino phenotype matches the advantage well. Representatively, the knockout mutant of the phytoene desaturase (*PDS*) gene, encoding an enzyme involved in the carotenoid biosynthesis pathway, exhibits an albino phenotype [28], so the *PDS* gene has been widely used as a target gene to verify genome editing efficiency in various plants, such as *Arabidopsis*, rice, cotton, chicory, grape, and watermelon [2,22,26,29,37,43]. Unfortunately, *pds* homozygous mutants cannot grow in soil and show albino and dwarf phenotypes with small rosette leaves. In addition, maintaining the *pds* mutant to the next generation is challenging due to the absence of bolting and flowering [28]. To eliminate such detrimental properties, in this study, the *PHOT2* gene, a blue light receptor, was verified as a target gene (Figure 2B). Phototropins are highly conserved across a wide range of plant species, from green algae to land plants [44,45]. In addition, the *phot2* mutant exhibits no avoidance response by which chloroplasts constantly accumulate at the cell surface under high-intensity blue light conditions [31,33]. Therefore, this distinct phenotype is easily observed by microscopy and observation with the naked eye [46]. In lettuce, CRISPR/Cas9-edited *phot2* mutants with biallelic mutations but not those with monoallelic mutations exhibited an accumulation response with the lack of an avoidance response under high-intensity blue light conditions (Figure 3C; Table 1). These phenotypes were easily observed in lettuce palisade mesophyll cells by confocal microscopy (Figure 3C and Figure 5D). In addition, the white band assay with leaves, which is often used to screen and evaluate chloroplast movement mutants, can observe chloroplast movement easily with the naked eye without expensive equipment [46]. Therefore, it is plausible that *PHOT2* could be a useful target gene in experiments to verify genome editing efficiency in various plants.

The chloroplast avoidance response induced under high-intensity light conditions is only mediated by phot2 [31,32,33]. The chloroplast avoidance response has an important physiological role in plant survival and in reducing photodamage under fluctuant light environments [31,47]. In other aspects, WT plants reach a maximum in photosynthetic efficiency at relatively low-intensity light compared with *phot2* plants because the chloroplast avoidance response is initiated at a range of light intensity lower than the light intensity requiring maximum photosynthetic ability. These characteristics of *phot2* mutants increased biomass by increasing the CO_2_ assimilation rate compared to WT in *Arabidopsis* [48]. Therefore, the usage of *phot2* characteristics could be a good strategy in crop biotechnology to improve the harvest index under controlled light environments such as plant factories.

## 4. Materials and Methods

### 4.1. Plant Materials and Growth Conditions

Red leaf lettuce (*Lactuca sativa* L. cv ‘Jeokchima’) (kindly provided by Prof. Tae-Sung Kim, Korea National Open University), a representative variety of lettuce that is a commonly consumed in Korea, was used in this study. Lettuce seeds were sterilized in 10% sodium hypochlorite solution (SAMCHUN, Korea) for 10 min and then washed 5 times with sterilized water. The sterilized seeds were sown on half-strength Murashige and Skoog (MS) medium (pH 5.7) containing 1% (*w/v*) sucrose and 0.8% (*w/v*) plant agar in plastic Petri dishes (90 mm in diameter, 20 mm in depth). Plates were stored at 4 °C in the dark for 2 days for simultaneous germination. Seedlings were grown under a photoperiodic condition (16-h light/8-h dark cycle of white light 100 μmol m^−2^ s^−1^) at 23 °C in a plant growth chamber.

### 4.2. Cloning of the LsU6-10 Promoter and Construction of the Binary Vectors

Information on lettuce *U6 small nuclear RNA* (*snRNA*) sequences was obtained using the gene search tool of the National Center for Biotechnology Information [(NCBI, https://www.ncbi.nlm.nih.gov/gene/?term=U6%20spliceosomal%20RNA%20Lactuca%20sativa (accessed on 22 October 2021)]. Ten candidate genes were pooled and identified using the *AtU6-26* gene (AT3G13855) as a query (Figure 1). The promoter region of the *LsU6-10* gene was amplified from lettuce gDNA using the LsU6-Fw (5’-CAAATGGATGGCATTCGAC-3’) and LsU6-Rv (5’-CGATAATGGATTCTGAGCTCG-3’) primer set and cloned into the pHAtC vector using the sites of the restriction enzyme *Aar*I (Figure 2A). 

The pHLsC vector was constructed by modifying the pHAtC vector (Addgene plasmid #78098) [40]. The *LsU6-10* promoter region was amplified by PCR with the LsU6-Fw-*Eco*RI (5’-AAGAATTCGGTATTGAGCAACTCCACAAG-3’) and LsU6-Rv-*Aar*I-*Xho*l (5’-AACTCGAGTCACCTGCCTCCGATAATGGATTCTGAGCTCG-3’) primer set, and the DNA fragment of the *LsU6-10* promoter was cloned into pHLsC by substituting the *AtU6* promoter using *Eco*RI and *Xho*l restriction enzyme sites of the pHAtC vector (see Figure 2A). To insert sgRNA361 into the pHAtC vector, two single-stranded oligonucleotides, At-phot2 sgRNA361-Fw (GATTGCCGATTATGTATGCTAGCAG) and phot2-sgRNA361-Rv (AAACCTGCTAGCATACATAATCGGC) were annealed and introduced into the pHAtC vector using the two *Aar*I restriction enzyme sites. Similarly, insertion of sgRNA361 into the pHLsC vector was carried out after two single-stranded oligonucleotides, Ls-phot2-sgRNA-Fw (TATCGCCGATTATGTATGCTAGCAG) and phot2-sgRNA-Rv, were annealed. The two T-DNA binary vectors, pHAtC and pHLsC harboring sgRNA361, were transformed into *Agrobacterium tumefaciens* strain GV3101 by the freeze–thaw method [49].

### 4.3. Agrobacterium-Mediated Lettuce Transformation

Cotyledons of 5-day-old red leaf lettuce seedlings were used for *Agrobacterium*-mediated transformation as previously described with some modifications [50]. Cotyledons were cut into 0.5 × 0.5 cm leaf discs with a razor, and the leaf discs (85–90 leaf discs) were incubated on cocultivation medium [MS media supplemented with 3% (*w/v*) sucrose, 0.5 mg/L 6-benzylaminopurine (BAP) (Sigma, St. Louis, MO, USA) and 0.1 mg/L α-naphthaleneacetic acid (NAA) (Sigma, St. Louis, MO, USA)] to induce calli at 23 °C for 2 days under a photoperiodic condition (16-h light/8-h dark cycle of white light 100 μmol m^−2^ s^−1^). 

*Agrobacteria* containing binary vectors, pHAtC-sgRNA361 and pHLsC-sgRNA361, were cultured until the OD600 reached 0.8 and were then resuspended in 20 mL of MS media solution containing 3% (*w/v*) sucrose, and the concentrations were finally adjusted to OD600 = 0.8. The leaf discs were inoculated with 20 mL of *Agrobacterium* solution for 30 min. The leaf discs were dried on sterilized filter paper after aqueous solutions were removed with sterilized filter paper and then cocultivated on cocultivation medium with 200 µM acetosyringone at 23 °C in the dark for 2 days. The leaf discs were transferred to selection medium (cocultivation medium supplemented with 15 mg/L hygromycin and 200 mg/L cefotaxime) to induce transformed calli for 1~2 months with repeated transfer to new selection medium every 2 weeks. The hygromycin-resistant calli were transferred to selection medium to induce shoot regeneration. Regenerated seedlings were grown on half-strength MS medium containing 3% (*w/v*) sucrose and 200 mg/L cefotaxime without phytohormone and antibiotics for rapid root regeneration for 2 weeks and were further grown on half-strength MS media containing 3% (*w/v*) sucrose, 15 mg/L hygromycin and 200 mg/L cefotaxime. The regenerated transgenic plants (R0 generation) were used for further molecular and physiological analyses.

### 4.4. PCR-Based Restriction Enzyme (PCR/RE) Digestion Assay

To carry out the PCR/RE digestion assay, genomic DNA (gDNA) was extracted from a leaf disc (1 cm × 1 cm) cut from 3-week-old lettuce using a DNA extraction buffer [200 mM Tris (pH 7.5), 250 mM NaCl, 25 mM ethylene-diamine-tetraacetic acid (EDTA, pH 8.0), 0.5% (*w/v*) sodium dodecyl sulfate (SDS)] as previously described [51]. The extracted gDNA (200 ng) was subjected to PCR amplification using Ex-Taq DNA polymerase (Takara, Japan). The PCR product amplified by the primer set phot2-44Fw (5’-CAAATGGATGGCATTCGAC-3’) and phot2-intron_Rv (5’-AAGAACACAGTAATTTAATCATCAG-3’) was digested with the restriction enzyme *Nhe*I at 37 °C for 3 h (see details in Figure 2). The digested DNA fragments were analyzed by 2% (*w/v*) agarose gel electrophoresis. 

### 4.5. Analysis of Chloroplast Photorelocation Movement

The chloroplast avoidance response was evaluated by observing chloroplast positionings under different light conditions (see below) as previously described [52]. For R0 transgenic lettuces, the leaves of transgenic plants that had roots in the regeneration medium were used for the experiment, and for WT and R1, R2 transgenic lettuces, the leaves of 2-week-old plants grown on half-strength MS medium were used for the experiment. The leaves were cut into appropriate sizes (0.5 cm × 0.5 cm), placed on a 0.5% (*w/v*) gellan gum plate, and treated with a high-intensity blue light (50 μmol m^−2^ s^−1^) for 2 h. Then, the leaves were treated with a fixation solution [20 mM 1,4-piperazinediethanesulfonic acid (PIPES), 5 mM MgCl_2_, 0.5 mM phenylmethylsulfonyl fluoride (PMSF), 1% (*w/v*) dimethyl sulfoxide (DMSO), and 2.5% (*w/v*) glutaraldehyde] for 30 min. Chloroplast positioning was observed by confocal microscopy (SP5, Leica, Wetzlar, Germany).

## Figures and Tables

**Table 1:** Genotype and phenotype of R0 phot2-At and phot2-Ls transgenic lines. *Nhe*I digestion: +, digested; −, not digested; +/−, partially digested. Mutation type: WT, no mutation; Mo, monoallelic mutation; Bi, biallelic mutation. Chloroplast positionings were examined after irradiation with a high-intensity blue light (50 μmol m^−2^ s^−1^) for 2 h Ac, accumulation; Av, avoidance. Indel patterns were summarized on the basis of data from *Nhe*I digestion and DNA sequencing, as shown in Figure 3A,B.

Transgenic Line (R0)	*Nhe*I Digestion	Mutation Type	Chloroplast Positioning	Indel Pattern
phot2-At1	+/−	Mo	Av	phot2-At1
phot2-At2	+/−	Mo	Av	phot2-At2
phot2-At3	−	Bi	Ac	phot2-At3
phot2-At4	−	Bi	Ac	phot2-At4
phot2-At5	+	WT	Av	WT
phot2-At6	+	WT	Av	WT
phot2-At7	+	WT	Av	WT
phot2-At8	+	WT	Av	WT
phot2-At9	+/−	Mo	Av	phot2-At1
phot2-At10	+	WT	Av	WT
phot2-At11	+	WT	Av	WT
phot2-At12	+/−	Mo	Av	phot2-At2
phot2-At13	+	WT	Av	WT
phot2-At14	+/−	Mo	Av	phot2-At1
phot2-At15	+/−	Mo	Av	phot2-At1
phot2-At16	+	WT	Av	WT
phot2-At17	−	Bi	Ac	phot2-At3
phot2-At18	+	WT	Av	WT
phot2-At19	−	Bi	Ac	phot2-At4
phot2-At20	+	WT	Av	WT
phot2-At21	+	WT	Av	WT
phot2-Ls1	+/−	Mo	Av	phot2-Ls1
phot2-Ls2	+/−	Mo	Av	phot2-Ls2
phot2-Ls3	+/−	Mo	Av	phot2-Ls3
phot2-Ls4	−	Bi	Ac	phot2-Ls4
phot2-Ls5	−	Bi	Ac	phot2-Ls5
phot2-Ls6	−	Bi	Ac	phot2-Ls6
phot2-Ls7	−	Bi	Ac	phot2-Ls7
phot2-Ls8	+	WT	Av	WT
phot2-Ls9	+/−	Mo	Av	phot2-Ls2
phot2-Ls10	−	Bi	Ac	phot2-Ls4
phot2-Ls11	+	WT	Av	WT
phot2-Ls12	+	WT	Av	WT
phot2-Ls13	−	Bi	Ac	phot2-Ls4
phot2-Ls14	+/−	Mo	Av	phot2-Ls3
phot2-Ls15	−	Bi	Ac	phot2-Ls6
phot2-Ls16	+	WT	Av	WT
phot2-Ls17	+/−	Mo	Av	phot2-Ls3
phot2-Ls18	+	WT	Av	WT
phot2-Ls19	−	Bi	Ac	phot2-Ls5
phot2-Ls20	+	WT	Av	WT
phot2-Ls21	+/−	Mo	Av	phot2-Ls2
phot2-Ls22	−	Bi	Ac	phot2-Ls6

**Table 2:** Editing efficiency and mutation types of CRISPR/Cas9-mediated *PHOT2* gene editing in R0 transgenic plants. Data were summarized from the data shown in Table 1.

Construct	Number of Transgenic Plants (R0 Generation)	Number of Transgenic Plants with Mutation	Mutation Frequency (%)	Mutation Type	
				Number of Monoallelic (%)	Number of Biallelic (%)
pHAtC	21	10	48	6 (29)	4 (19)
pHLsC	22	16	73	7 (32)	9 (41)

## Data Availability

The plant materials are available from the corresponding author (Sam-Geun Kong) upon reasonable request.
